# The best solution down the line: an observational study on taurolidine- versus citrate-based lock solutions for central venous catheters in hemodialysis patients

**DOI:** 10.1186/s12882-021-02519-3

**Published:** 2021-09-13

**Authors:** Sonja van Roeden, Mathijs van Oevelen, Alferso C. Abrahams, Friedo W. Dekker, Joris I. Rotmans, Sabine C. A. Meijvis, on behalf of the DUCATHO study group, J. A. Bijlsma, K. E. A. van der Bogt, A. van de Brug, C. E. Douma, E. J. Hoorn, D. H. T. IJpelaar, M. J. Krol-van Straaten, K. W. Mui, J. H. M. Tordoir, H. H. Vincent, N. Zonnebeld

**Affiliations:** 1 Department of Nephrology and Hypertension, University Medical Center Utrecht, Heidelberglaan 100, 3584 CX Utrecht, The Netherlands; 2 Department of Internal Medicine, Leiden University Medical Center, Leiden, The Netherlands; 3 Department of Clinical Epidemiology, Leiden University Medical Center, Leiden, The Netherlands

**Keywords:** Central venous catheter, Hemodialysis, Lock, Lock solution

## Abstract

**Introduction:**

To prevent infection and thrombosis of central venous catheters (CVCs) in hemodialysis patients, different CVC lock solutions are available. Taurolidine-based solutions and citrate in different concentrations are frequently used, but no definite conclusions with regard to superiority have been drawn.

**Methods:**

In this retrospective, observational, multicenter study, we aimed to assess the risk for removal of CVC due to infection or catheter malfunction in hemodialysis patients with CVC access for different lock solutions: taurolidine, high-concentrated citrate (46.7%) and low-concentrated citrate (4 or 30%). A multivariable Cox-regression model was used to calculate hazard ratio’s (HR).

**Results:**

We identified 1514 patients (median age 65 years, 59% male). In 96 (6%) taurolidine-based lock solutions were used. In 1418 (94%) citrate-based lock solutions were used (high-concentrated 73%, low-concentrated 20%). Taurolidine-based lock solutions were associated with a significantly lower hazard for removal of CVC due to infection or malfunction combined (HR 0.34, 95% CI 0.19–0.64), and for removal of CVC due to infection or malfunction separately (HR 0.36, 95% CI 0.15–0.88 and HR0.33, 95% CI 0.14–0.79). High-concentrated citrate lock solutions were not associated with a decreased hazard for our outcomes, compared to low-concentrated citrate lock solutions.

**Conclusion:**

Removal of CVC due to infection or catheter malfunction occurred less often with taurolidine-based lock solutions. We present the largest cohort comparing taurolidine- and citrate-based lock solutions yet. However, due to the retrospective observational nature of this study, conclusions with regard to superiority should be drawn with caution.

## Background

In patients with kidney failure, arteriovenous fistulas (AVF) are first choice option for vascular access for hemodialysis [1]. However, AVF placement may not always be feasible prior to the start of hemodialysis. In case of acute kidney injury, sudden deterioration of chronic kidney disease, AVF occlusion or maturation failure, alternative vascular access is needed [1].

Central venous catheters (CVC) provide easy and instant vascular access. However, CVC are associated with an increased risk for infectious complications and catheter malfunction, when compared to AVF [2]. Infections are the leading cause of catheter removal and contribute significantly to morbidity and mortality in hemodialysis patients [2, 3]. The risk for bloodstream infections is over fifteen-fold increased in patients with CVC access, compared to patients with AVF access [2]. Besides the risk for infectious complications, catheter malfunction is a frequent problem in hemodialysis with CVC as vascular access. Catheter malfunction may be due to thrombosis, malposition or fibrin sheet formation, resulting in impaired flow. Up to 50% of all CVC fail within 1 year after placement and failure is associated with morbidity and increase in costs [4, 5]. Moreover, mortality is higher in patients with CVC in comparison to those with grafts and fistulas, although this may be due to other factors such as the general condition and comorbidity [5].

To prevent infection and thrombosis, different CVC lock solutions are available and used. In all patients with CVC for hemodialysis, lock solutions are indicated and used. Choice for lock solution type may be determined by the treating physician, since there is no hard evidence on superiority of any of the available options, according to the guidlines [6]. For lock solutions containing antibiotics, concerns with regard to antibiotic resistance have been raised [7]. Therefore, antibiotic-free alternatives such as taurolidine- and citrate-based lock solutions with both antimicrobial and antithrombotic properties are frequently used. Both taurolidine and citrate have antimicrobial activity through disruption of the bacterial cell membrane, by causing a chemical reaction. Taurolidine leads to introduction of methylol groups in the cell membrane, whereas citrate leads to chelation of magnesium, both leading to disruption of cell membrane integrity [8, 9]. The antithrombotic properties of taurolidine rely on decreasing the activity of coagulation factors I, VIII, XI and XII, while citrate prevents platelet activation by chelation of calcium [10, 11]. Currently, it is unclear what the optimal lock solution for prevention of CVC-related infections and thrombosis is. Taurolidine- and citrate-based lock solutions have been found to be superior to heparin-based lock solutions in terms of CVC-related bloodstream infections (BSI) and equally effective in prevention of CVC-associated thrombosis [12–14]. No definite conclusions with regard to superiority of citrate or taurolidine-based lock solutions for prevention of CVC-related BSI and thrombosis have been drawn. Studies that compared the efficacy of taurolidine- and citrate-based lock solutions are scarce. In one small trial, taurolidine-based lock solutions were more effective in preventing CVC-related infections and dysfunction compared to citrate-based lock solutions with a low concentration (4%) [15]. On theoretical grounds, citrate-based lock solutions with higher concentrated citrate could be more effective. However, concerns with regard to safety for citrate-based lock solutions with higher concentrations have been raised, while superiority remains unproven [16–20]. The Food and Drug Administration discourages use of highly concentrated citrate after an accidental fatal incident with intravenous administration of the lock solution [20]. The actual incidence of citrate toxicity is unknown.

Altogether, there is an urgent need for comparison taurolidine- and citrate-based lock solutions in different concentrations. We aimed to evaluate the efficacy of taurolidine- and citrate-based lock solutions in reducing the risk of infectious complications and catheter malfunction in a large cohort of patients with CVC for hemodialysis. Moreover, we describe characteristics and the incidence of CVC-related infections in a large cohort of patients with CVC for hemodialysis.

## Methods

### Study design and data collection

We performed a retrospective observational multicenter cohort study to assess the risk of infection and catheter malfunction during use of taurolidine- and citrate-based lock solutions in hemodialysis patients with CVC access [21]. All patients are derived from the DUCATHO database, clinical data of all adult patients in whom a CVC was inserted between January 1st 2012 and December 31st 2016 for hemodialysis from 12 participating hospitals (academic and non-academic) in the Netherlands are stored [21]. Design of this database was approved by the Medical Ethics Committee of the University Medical Center Utrecht (METC Utrecht) [21]. Data were collected from electronical patient records. Obtaining informed consent was waived by the Medical Ethics Committee since data were collected and processed anonymously. If patients objected against use of their medical record for research purposes, if a CVC was used for continuous venovenous hemofiltration or if the patient underwent hemodialysis in a non-participating center during the CVC period, they were not included in the database. The study was performed in line with STROBE guidelines for observational cohort studies.

### Outcomes

The primary outcome of this study was the hazard ratio for a combined endpoint consisting removal of CVC due to either infection or due to catheter malfunction. The secondary outcomes of this study were the hazard ratios for (1) removal of CVC due to infection or (2) removal of CVC due to catheter malfunction separately. Only events occurring during the first CVC of each patient were studied in formal analysis, since the chance of events for consecutive CVC may also be dependent on the occurrence of events during earlier CVC. Additional to our primary and secondary outcomes, the incidence and incidence rates of proven CVC-related BSI with corresponding pathogens were reported in the entire cohort. A proven CVC-related BSI was defined as the presence of a positive blood culture, associated with a raised systemic inflammatory response, and absence of clinical signs of a non-CVC-related source of infection.

### Intervention

The lock solutions that were studied in this analysis were taurolidine-based lock solutions (consisting of taurolidine combined with citrate 4% and/or 500 U heparin/ml), high-concentrated citrate lock solutions (46.7%) and low-concentrated citrate lock solutions (with either 4% or 30% citrate lock solutions). Choice for lock solution is non-standardized and based on the preference of hemodialysis center and treating physician (in accordance with international guidelines) [6]. The lock solution started at insertion of CVC was registered. If the type of lock solution was altered during insertion of one CVC, this has not been registered in our database. However, we believe that the number of patients with alteration of lock solution type is limited.

### Statistical methods

Data were stored in an SPSS (version 21.0) database. Descriptive data were generated in SPSS. All statistical analyses were performed in R studio (version 3.2.2). For survival analysis, patients were censored after the first event, since consecutive events within patients are not independent. Multivariable analysis was performed with a Cox proportional hazards model. Cause specific hazard ratios (HR) with 95% confidence intervals (CI) for primary and secondary outcomes were calculated for different lock solutions. On theoretical grounds, age and gender were identified as potential confounders and entered as covariates in the model. No random effects were fitted in the model to correct for correlation in data of patients from the same hospitals, since the lock solution type was strongly correlated to hospital site. The proportional hazards assumption was verified with both formal tests and graphically, using Schoenfeld residuals. The Cox regression models were fitted with the “cmprsk” and “survival” packages. *P*-values ≤0.05 were considered statistically significant. The incidence of BSI was reported for all CVC as number of events per 1000 CVC days. Patients with missing data with regard to the determinant, covariates or outcome were excluded from formal analysis. Numbers of missing data were reported in the results section of this article.

### Sensitivity analyses

Due to the observational, non-standardized nature of this study, extraneous factors may influence the results, leading to residual confounding. To explore the possibility of such factors explaining our findings, sensitivity analyses were performed. Since most patients using taurolidine-based lock solutions originated from one specific center, the main analysis (a multivariable Cox proportional hazards model) was repeated within this specific center only. Also, the analysis was repeated with a frailty term integrated in the model to correct for correlation in data of patients from the same center (assuming a Gaussian distribution of the frailty term). Furthermore, the analysis was repeated for patients with jugular CVC only, to explore if CVC insertion site influenced the outcomes. Also, the analysis was repeated with stratification for CVC type (tunneled, precurved untunnelled or straight untunnelled), to explore if CVC type influenced the outcomes of this study. Finally, we studied 30% citrate lock-solutions and 46.7% citrate lock-solutions together (eliminating 4% citrate lock-solutions) for comparison to taurolidine-based lock solutions, to investigate if the definition of our groups influence the outcomes of this study.

## Results

In total, we identified 1603 unique patients. In 40 patients, heparin-based lock solutions were used. In 49 patients, data with regard to lock solution type were missing. These patients were excluded from analysis, leaving 1514 patients with a total of 139,217 CVC days. Median age for all patients was 65 years (IQR 53–74), 59% of patients (*n* = 942) were male. For 640 patients, hemodialysis was initiated in an acute setting (40%). For 1514 patients, the lock solution of the first line consisted of taurolidine in 96 patients (total 16,625 CVC days), high-concentrated citrate in 1110 patients (96,980 CVC days) and low-concentrated citrate in 308 patients (25,612 CVC days). See Table 1 for details of patients with citrate or taurolidine-based lock solutions.

**Table 1:** Baseline characteristics of patients and CVC characteristics of first line

	All	Taurolidine^a^	High- concentrated citrate	Low-concentrated citrate
**N (%)**	1514	96 (6)	1110 (73)	308 (20)
**Age (median, IQR)**	65 (53–74)	58 (42–69)	66 (54–75)	63 (53–73)
**Male gender (%)**	893 (59)	59 (61)	649 (58)	185 (60)
**Days of CVC in situ (median, IQR)**	37 (11–120)	102 (27–210)	35 (9–112)	35 (13–118)
**Acute start of dialysis (%)**	605 (40)	30 (31)	421 (38)	154 (50)
**History of diabetes mellitus (%)**	555 (37)	34 (35)	421 (38)	100 (32)
**Use of immunosuppressive medication (%)**	411 (27)	21 (22)	296 (27)	94 (31)
**Type of CVC (%)**				
Tunneled	404 (27)	39 (41)	301 (27)	64 (21)
Non-tunneled, precurved	624 (41)	51 (53)	459 (41)	114 (37)
Non-tunneled, straight	461 (30)	6 (6)	330 (30)	125 (41)
**Concentration lock (%)**				
4%	55 (4)	–	–	55 (18)
30%	253 (17)	–	–	253 (82)
46.7%	1110 (73)	–	1110 (100)	–
**Insertion site (%)**				
Jugular	1182 (78)	89 (93)	862 (78)	231 (75)
Subclavian	37 (2)	4 (4)	27 (2)	6 (2)
Femoral	276 (18)	3 (3)	205 (18)	68 (22)
**CVC lumen > 14 fr (%)**	675 (45)	36 (38)	484 (44)	155 (50)
**Removal of CVC due to infection (%)**	156 (10)	6 (6)	120 (11)	30 (10)
**Removal of CVC due to patency problem (%)**	163 (11)	6 (6)	119 (11)	38 (12)
**Death (%)**	407 (27)	24 (25)	329 (30)	54 (18)
due to (any) infection	83 (5)	4 (4)	62 (6)	17 (6)
cessation of dialysis	108 (7)	1 (1)	93 (8)	14 (5)

^a^92 consisted of taurolidine and heparine500, 4 consisted of taurolidine only

### Primary and secondary outcomes

**Fig. 1:**
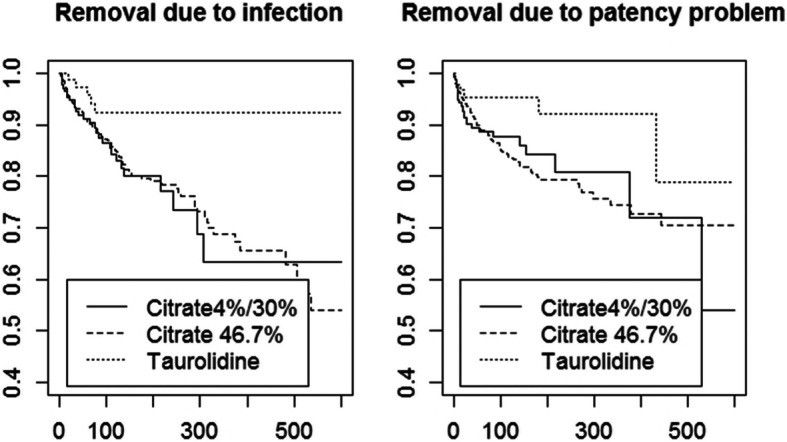
Kaplan-Meier survival curves for the primary and secondary endpoint per lock solution category

CVC was removed due to infection in 156 patients (10%, median time to removal 52 days) and due to catheter malfunction in 163 patients (11%, median time to removal 28 days) with taurolidine- or citrate-based locks. The numbers of events for different lock solutions are summarized in Table 2 and univariable survival analysis is shown in Fig. 1*.* In multivariable analysis, taurolidine-based lock solutions were associated with a significantly lower hazard for removal of CVC due to infection or catheter malfunction (combined) with a HR of 0.34 (95% CI 0.19–0.64). When studying both secondary endpoints separately, taurolidine-based lock solutions were also associated with a significantly lower hazard for removal of CVC due to infection (HR 0.36, 95% CI 0.15–0.88) and removal of CVC due to catheter malfunction (HR 0.33, 95% CI 0.14–0.79) compared to low-concentrated citrate lock solutions (4% or 30%). High-concentrated citrate lock solutions were not associated with a decreased hazard for the primary and secondary outcome compared to low-concentrated citrate lock solutions (Table 2*)*.

**Table 2:** Hazard ratio’s for primary and secondary outcomes

Endpoint / Lock type	Events	Patients	HR	95%CI	***p***-value
**Primary outcome**					
Citrate 4% or 30%	68	308	Ref.		
Citrate 46.7%	239	1110	0.96	0.73–1.26	0.76
Taurolidine	12	96	0.34	0.19–0.64	< 0.001
**Secondary outcome – infection**					
Citrate 4% or 30%	30	308	Ref.		
Citrate 46.7%	120	1110	1.10	0.74–1.64	0.64
Taurolidine	6	96	0.36	0.15–0.88	0.02
**Secondary outcome – catheter malfunction**					
Citrate 4% or 30%	38	308	Ref.		
Citrate 46.7%	119	1110	0.85	0.59–1.23	0.39
Taurolidine	6	96	0.33	0.14–0.79	0.01

Ref. indicates reference catgory

### Sensitivity analysis

When studying the hazard for our primary outcome within the one center accounting for 94% of patients using taurolidine-based lock solutions (total *n* = 178), findings were similar to the initial analysis (HR 0.36, 95% CI 0.17–0.76 for taurolidine-based lock solutions). When studying the hazard for our primary outcome in all patients after correction for center, findings were similar to the initial analysis again (HR 0.34, 95% CI 0.15–0.76 for taurolidine-based lock solutions). The estimates did not change when studying jugular CVC only (HR 0.39, 95% CI 0.20–0.76 for taurolidine-based lock solutions) or after stratification for CVC type (tunneled, precurved or straight) (HR 0.39, 95%CI 0.21–0.74 for taurolidine-based lock solutions). Furthermore, the estimates did not change when regrouping citrate 30 and 46.7% lock solutions together (and eliminating citrate 4% lock solutions), and comparing this group to taurolidine lock solutions (HR 0.36, 95% CI 0.20–0.64).

### CVC-related BSI

We explored the number of CVC-related BSI with causative pathogens for all consecutive lines (so not first lines only) with taurolidine-based and citrate-based lock solutions. In all 1603 patients, 2749 CVC were placed with a total of 271,295 CVC days. Overall incidence rate of proven catheter-related BSI was 0.83 per 1000 CVC days. The incidence rate of proven catheter-related BSI was lowest for taurolidine-based lock solutions (0.37 per 1000 CVC days) compared to citrate-based lock solutions (0.89 per 1000 days for high-concentrated citrate and 0.83 per 1000 days for low-concentrated citrate). More details on causative pathogens are summarized in Table 3.

**Table 3:** Incidence of infections and corresponding pathogens

	All	Taurolidine	High- concentrated citrate	Low-concentrated citrate
**Number of patients (any CVC, %)**^a^	1603	129 (8)	1132 (71)	317 (20)
**Number of CVC in total (%)**	2749	211 (8)	1840 (67)	490 (18)
**Total number of CVC days (median, IQR)**	271,295	40,671	166,819	43,533
**Number of proven catheter-related BSI**^b^**(%)**	225	15 (12)	149 (13)	36 (11)
**Incidence rate of BSI per 1000 catheter days**^c^	0.83	0.37	0.89	0.83
**Number of proven catheter-related BSI for femoral CVC (%)**	16	2 (10)	9 (3)	5 (5)
**Incidence rate of BSI per 1000 catheter days for femoral CVC**	1.77	0.80	1.70	4.03
**Type of pathogen during BSI**^d^				
Coagulase negative staphylococci (%)	72 (32)	5 (33)	46 (31)	11 (31)
*S. aureus* (%)	61 (27)	2 (13)	45 (30)	9 (25)
Gram-negative (%)	43 (19)	4 (27)	30 (20)	6 (17)
Gram-positive (%)	40 (18)	3 (20)	24 (16)	9 (25)
Yeast (%)	2 (< 1)	–	–	1 (3)

^a^different lock solutions within patients during different CVC possible. In this table, all consecutive CVC are taken in to account and patients who used citrate or taurolidine locks later on (not only first) lines were also included)

^b^14 CVC with > 1 episode of BSI: 9 with citrate lock and 1 with taurolidine lock

^c^for all lines

^d^multiple pathogens per episode possible. Unknown pathogen in 14 positive cultures

### Missing data

Data with regard to lock solution type were missing in 3% (49 / 1603). Data with regard to the outcomes were missing in < 0.001% (1 / 1603). Data with regard to covariates were missing in < 0.01% (6 / 1603).

## Discussion

In this retrospective, observational cohort study, removal of CVC due to infection or catheter malfunction occurred less often in hemodialysis patients with taurolidine-based lock solutions compared to citrate-based solutions. We did not observe any differences between high-concentrated citrate lock solutions and low-concentrated lock solutions. Our findings suggest that taurolidine-based lock solutions may be superior to citrate-based lock solutions in terms of infections and catheter malfunction.

In earlier studies, conflicting results were found with regard to the optimal lock solution. It has been established that taurolidine- and citrate-based lock solutions are superior to heparin-based lock solutions in terms of CVC-related BSI and equally effective in prevention of CVC-associated thrombosis [12–14]. However, results of studies comparing taurolidine and citrate are scarce. Taurolidine-based lock solutiones have been observed to be more effective in preventing CVC-related infections and catheter dysfunction compared to low-concentrated (4%) citrate lock solutions in one randomized controlled trial with 106 patients. The study was criticized for having a relatively high rate of catheter-related bacteremia, which may be due to suboptimal hygiene protocols [22]. Moreover, the effect of taurolidine was mainly based on a reduction in catheter-related bacteremia caused by gram-negatives, whilst gram-positives are in general the most frequent causative micro-organisms of catheter-related bacteremia [22].

Besides the effect of lock solution type, we presented the number of proven CVC-related BSI in our cohort. The CVC-related BSI per 1000 CVC days in our cohort was < 1, which is below the targeted CVC-related BSI rate during hemodialysis in the Netherlands [23]. We observed a clear difference in the incidence of CVC-related BSI for different lock solutions, which is coherent with the outcomes of the multivariable Cox regression proportional hazards model, that showed that taurolidine-based locks are associated with a lower risk of infection.

It appeared that CVC-related BSI in patients with taurolidine-based locks were less often caused by *S. aureus,* and slightly more often by gram-negative pathogens. In vitro, both citrate and taurolidine-based lock solutions are active against a large spectrum of gram-negative and gram-positive micro-organisms [24, 25]. In vivo however, it has been shown that taurolidine-citrate-heparin catheter lock solutions are associated with a decreased risk for *Staphylococcal* bloodstream infections [13]. From the current study, no hard conclusions can be drawn with regard to the clinical efficacy in prevention of CVC-related BSI caused by specific pathogens, since numbers of events caused by specific pathogens were too small.

The major strength of the current study is the large number of patients studied, included from 12 academic and non-academic hospital sites throughout the Netherlands over 5 years. Apart from one small RCT [15], there are currently no clinical data available on the efficacy of taurolidine-based lock solutions versus citrate-based lock solutions. Furthermore, the topic studied is highly relevant: to our knowledge, no systematic comparison of different concentrations of citrate and taurolidine-based lock solutions has been performed yet. Finally, we chose a hard and unambiguous endpoint: removal of CVC due to infection or catheter malfunction.

Our study has drawbacks as well. Due to the observational nature of this study, decisions with regard to lock solution type were unstandardized. This may lead to confounding by indication: clinicians may provide more proactive and preventive care to the most vulnerable patients, leading to underestimation of the effect of such a strategy. Second, we observed that lock solution type is depending on hospital site. The choice for lock solution type probably does not depend on patient characteristics but on routine practice, that differs between centers. Such ‘center effect’ might explain part of the lower hazard for our outcomes in patients treated with taurolidine-based locks. Indeed, most patients using taurolidine-based lock solutions were included in one center. In this center, approximately half of patients used taurolidine-based lock solutions, the other half used citrate lock solutions: prior to 2014 patients used citrate lock solutions predominantly, while patients treated after 2014 used taurolidine-based lock solutions predominantly. We studied the effect of lock solution type within this center and after correction for center as a sensitivity analysis, to evaluate if the lower hazard for infection or catheter malfunction remains: results were comparable to the analysis of the entire cohort. If this specific center would have changed their hygiene policy simultaneously with their choice of lock solution, this may results in bias. However, the fact that the use of taurolidine-based lock solutions was associated with both secondary endpoints separately, suggests otherwise (since hygiene may influence the risk for infection, but not the risk for catheter malfunction). Nevertheless, validation of these findings in a randomized controlled setting is desirable.

Moreover, any change of lock solution type within patients during insertion of one CVC, has not been registered in our dataset. This may distort the outcomes of our study. However, since choice of lock solution type is mainly depending on hemodialysis center and treating physician, the number of alteration within patients is probably very low.

## Conclusions

In conclusion, removal of CVC due to infection or catheter malfunction occurred less often in hemodialysis patients with taurolidine-based lock solutions. High-concentrated citrate lock solutions (46.7%) were not superior to lower concentrated citrate lock solutions (30% or 4%). Due to the retrospective observational nature of this study, definite conclusions with regard to superiority should be drawn with caution: validation of our findings in a prospective and standardized setting, ideally in a randomized controlled trial, is desirable.

## Data Availability

The datasets generated during and analyzed during the current study are not publicly available, in line with DUCATHO study group policies, but are available from the corresponding author on reasonable request.
